# Inflammatory myofibroblastic tumor of the thigh without bone involvement: a case report

**DOI:** 10.1186/1477-7819-12-208

**Published:** 2014-07-15

**Authors:** Jun Lin, Hao Liu, Yin Zhuang, Peng Yang, Yifei Zheng, Yan Yang, Huilin Yang

**Affiliations:** 1 Department of Orthopedic Surgery, The First Affiliated Hospital of Soochow University, 188 Shizi Street, Suzhou, Jiangsu 215006, China

**Keywords:** Inflammatory myofibroblastic tumor, Pseudotumor, Magnetic resonance imaging

## Abstract

Inflammatory myofibroblastic tumors are rare, and those located in the extremities without bone involvement are even rarer. We present the case of a 61-year-old Chinese male patient with an inflammatory myofibroblastic tumor of the right thigh. It was excised and a histopathologic examination revealed an inflammatory myofibroblastic tumor. This case is presented by virtue of its rare location.

## Background

Inflammatory myofibroblastic tumor (IMT) is an uncommon benign neoplasm with partially invasive behavior and a tendency to recur [1]. IMTs are commonly found in the lung [2-4]. Extrapulmonary IMTs occur in nearly every site in the body, however, it is unusual for IMTs to exist in the lower extremities without bone involvement [1,3-5]. Herein, we illustrate an unusual case of an IMT of the thigh without bone involvement.

## Case presentation

**Figure 1:**
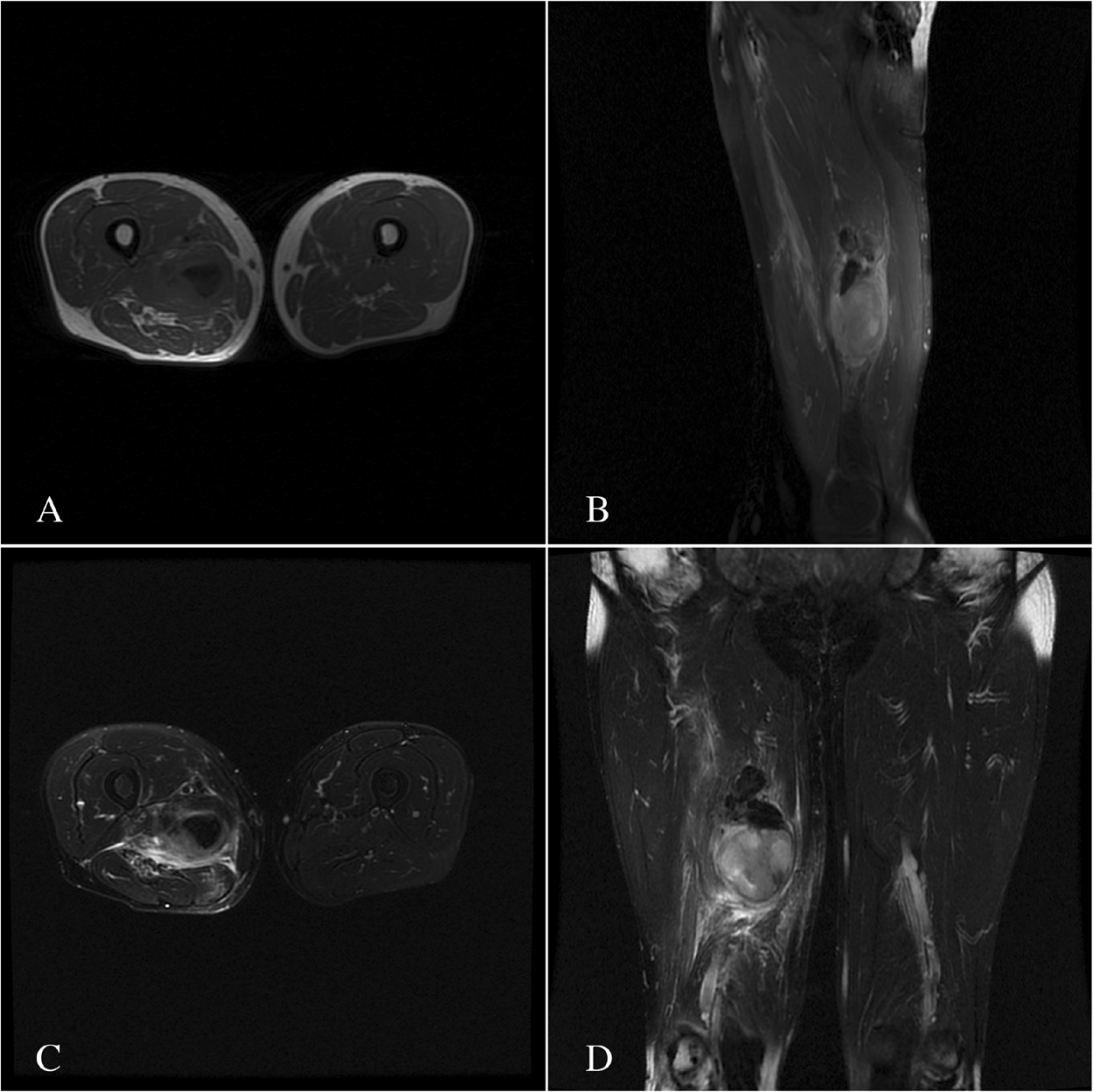
**T1-weighted and STIR images in MRI for the lesion.** Axial and sagittal T1-weighted image **(A, B)** show an inhomogeneously isointense mass in the posterior thigh. The lesion demonstrates inhomogeneously hyperintense on axial and coronal STIR images **(C, D)**.

**Figure 2:**
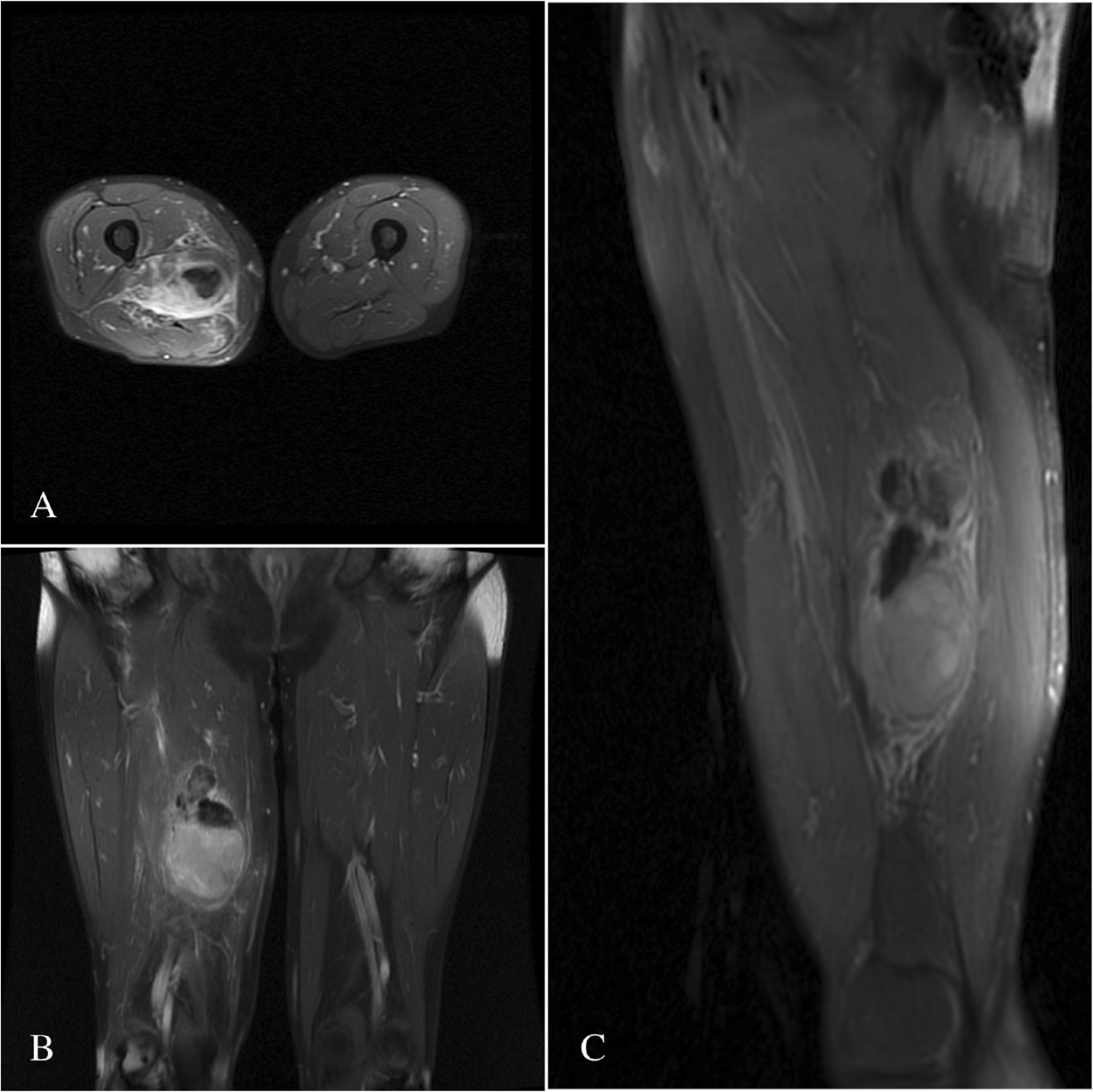
**Enhanced T1-weighted and T2-weighted images in MRI for the lesion.** Axial and coronal enhanced T1-weighted images show moderate enhancement **(A, B)**. Sagittal T2-weighted image **(C)** shows an inhomogeneously isointense mass in the posterior thigh.

**Figure 3:**
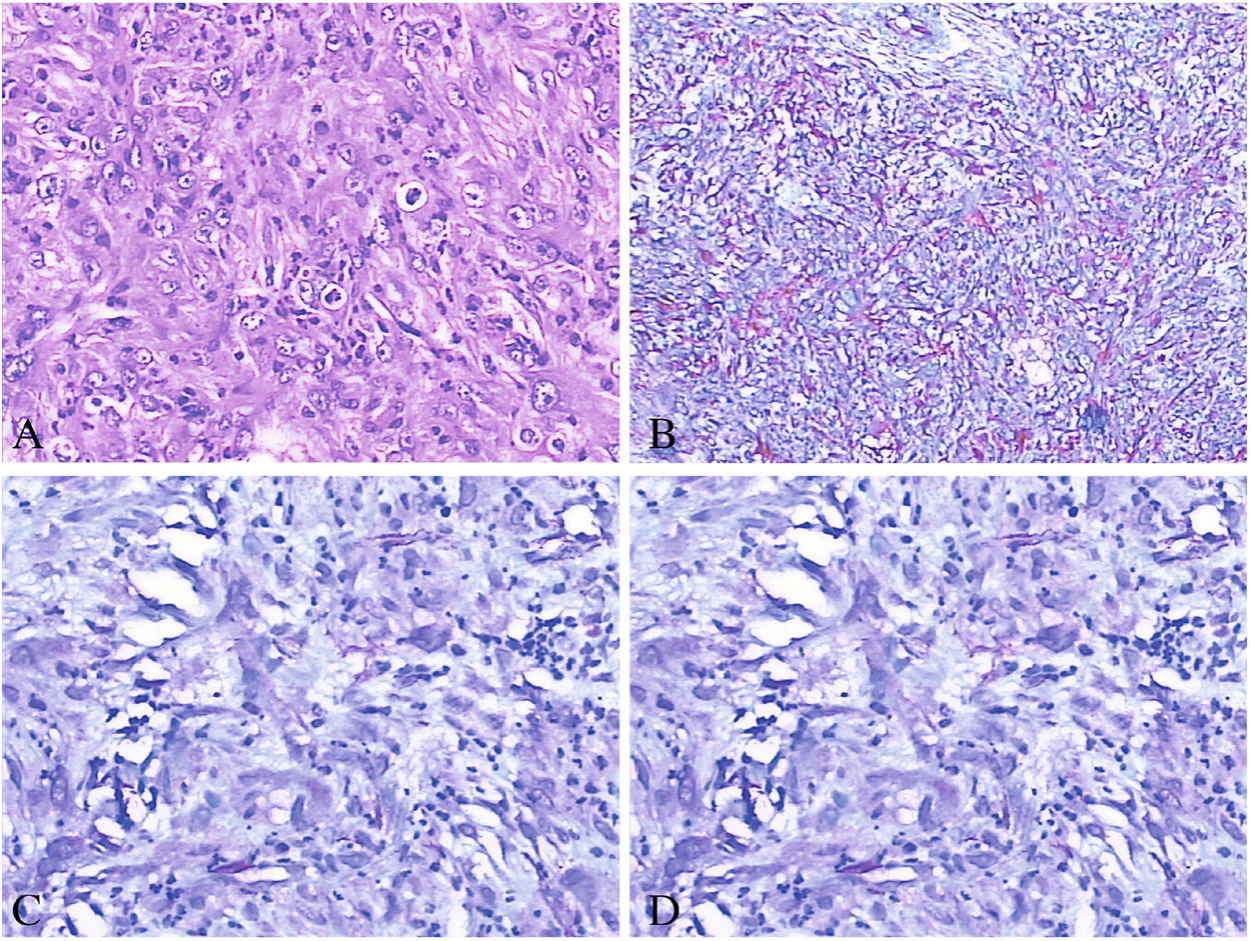
**Photomicrograph by hematoxylin-eosin (HE) and immunohistochemistry staining for tumor.** Photomicrograph showing proliferation of eosinophilic spindle cells with numerous inflammatory cells including lymphocytes and few granulocytes. [HE, original magnification, ×200] **(A)**. Immunohistochemistry revealed tumor cell immunoreactivity for vimentin **(B)**, smooth muscle actin (SMA) **(C)**, and CD68 **(D)** (original magnification, ×200).

A 61-year-old Chinese male patient was referred to our service with a history of severe pain in his right thigh for the most recent 18 months. There was no history of trauma. A physical examination revealed a moderately hard, immovable, and painful 150 × 100 mm mass of the posterior thigh.A magnetic resonance imaging (MRI) scan revealed a lesion in the right side of the adductor magnus muscle. The lesion was inhomogeneously isointense on the T1-weighted images (Figure 1A, B) and the T2-weighted image (Figure 2C), and inhomogeneously hyperintense on the short TI inversion-recovery (STIR) images (Figure 1C, D) with marked contrast enhancement after administration of gadolinium (Figure 2A, B). Further examinations of contrast-enhanced MRI scans of the brain and CT scans of the chest and abdomen showed no evidence of metastases.The histologic findings of the biopsy performed before the operation suggested a spindle cell tumor with mild atypia. Subsequently, an excision of the tumor was performed. A microscopic examination showed spindle tumor cells arranged in an irregular pattern with variable cell density. The tumor was infiltrated by some lymphocytes and a few neutrophil granulocytes (Figure 3A). The spindle cells showed mild atypia and no mitoses were observed. It was revealed on immunohistochemistry that the tumor showed positive immunoreactivity for vimentin, α-smooth muscle actin (α-SMA), and CD68 (Figure 3B, C, D), but was negative for anaplastic lymphoma kinase (ALK), S-100, CD34, CD117, kinesin-like protein-1 (KP-1), myelin basic protein (MBP), and desmin. The patient’s recovery was uneventful.

## Discussion

Since two cases of spindle benign tumor of lung were reported by Brunn in 1939 [6], increasing amounts of researchers have paid close attention to this intermediate type of tumor with low potential malignancy. The World Health Organization (WHO) classification of tumors of soft tissue and bone currently defines IMT as a distinctive neoplasm composed of myofibroblastic and fibroblastic spindle cells accompanied by an inflammatory infiltrate of plasma cells, lymphocytes, and/or eosinophils [7]. Most cases of IMT have been found in the lung, orbit, mesentery and omentum, and gastrointestinal and genitourinary tracts [1] in recent years. Several IMTs [8,9] often invade bone tissue with systemic symptoms in orthopedic surgery. To the best of our knowledge, this is the first reported case of IMT in the lower extremities without bone involvement and metastasis to other organs.

Extrapulmonary IMTs are more common in children and young adults with an average age of 10-years-old, and the incidence of men and women is 1 to 1.4 [2-4], of which the etiology is unknown. Though reports of postsurgical, posttraumatic, and postinfectious cases have prompted speculation that the process is initially reactive, these patients will fall into an overtly neoplastic disease category [5].

Patients suffering from IMTs of the thigh have no systemic symptoms (such as anemia, unexplained fever, or weight loss) or laboratory abnormalities. Besides MRI scans, histomorphology and immunohistochemical staining is the most helpful tool in the diagnosis of IMTs. Furthermore, cytogenetic studies have shown clonal rearrangements of the short arm of chromosome 2, involving the ALK receptor tyrosine kinase locus region, in up to 50% of soft tissue IMTs [2,10,11]. ALK, a surrogate for ALK gene rearrangement, has been suggested as a good immunohistochemical marker for IMT. However, ALK-negative IMTs are indistinguishable histologically from ALK-positive ones [12]. Our case was negative for ALK. In recent reports, it has been shown that dedifferentiated liposarcoma can have prominent inflammatory myofibroblastic tumor-like features with expressions of MDM2 and CDK4 for identification [13,14]. Both markers MDM2 and CDK4 were not detected in our case. We suggest that the presence or absence of a well-differentiated liposarcoma component and expressions of MDM2 and CDK4 should be considered in the diagnosis of IMTs and requires further research.

## Conclusions

In summary, we have reported an additional case of IMT of the lower extremities without bone involvement. This was treated with excision, and clinical and histological features were consistent with a benign lesion.

## Consent

Written informed consent was obtained from the patient for publication of this case report and any accompanying images. A copy of the written consent is available for review by the Editor-in-Chief of this journal.

## Abbreviations

ALK: Anaplastic lymphoma kinase; HE: Hematoxylin-eosin; IMT: Inflammatory myofibroblastic tumor; KP-1: Kinesin-like protein-1; MBP: Myelin basic protein; MRI: Magnetic resonance imaging; STIR: Short TI inversion-recovery; SMA: Smooth muscle actin; WHO: World Health Organization.

## Competing interests

The authors declare that they have no competing interests.

## Authors’ contributions

JL and HL contributed equally to this work. JL, HL and YZ contributed to the drafting and final revisions of the manuscript. PY, YFZ and YY contributed to the drafting of the manuscript. All of the authors approved the final version of the manuscript.
