# Correction: Inflammatory myofibroblastic tumor of the thigh without bone involvement: a case report

**DOI:** 10.1186/s12957-023-03158-8

**Published:** 2023-09-05

**Authors:** Jun Lin, Hao Liu, Yin Zhuang, Peng Yang, Yifei Zheng, Yan Yang, Huilin Yang

**Affiliations:** Department of Orthopedic Surgery, The First Affiliated Hospital of Soochow, University, 188 Shizi Street, Suzhou, 215006 Jiangsu China

**Correction: World J Surg Onc 12, 208 (2014)**

**https://doi.org/10.1186/1477-7819-12-208**

Following the publication of the original article [1], the author reported that Fig. 3D is a repetition of Fig. 3C. The correct figure is included here.

**Fig. 3:**
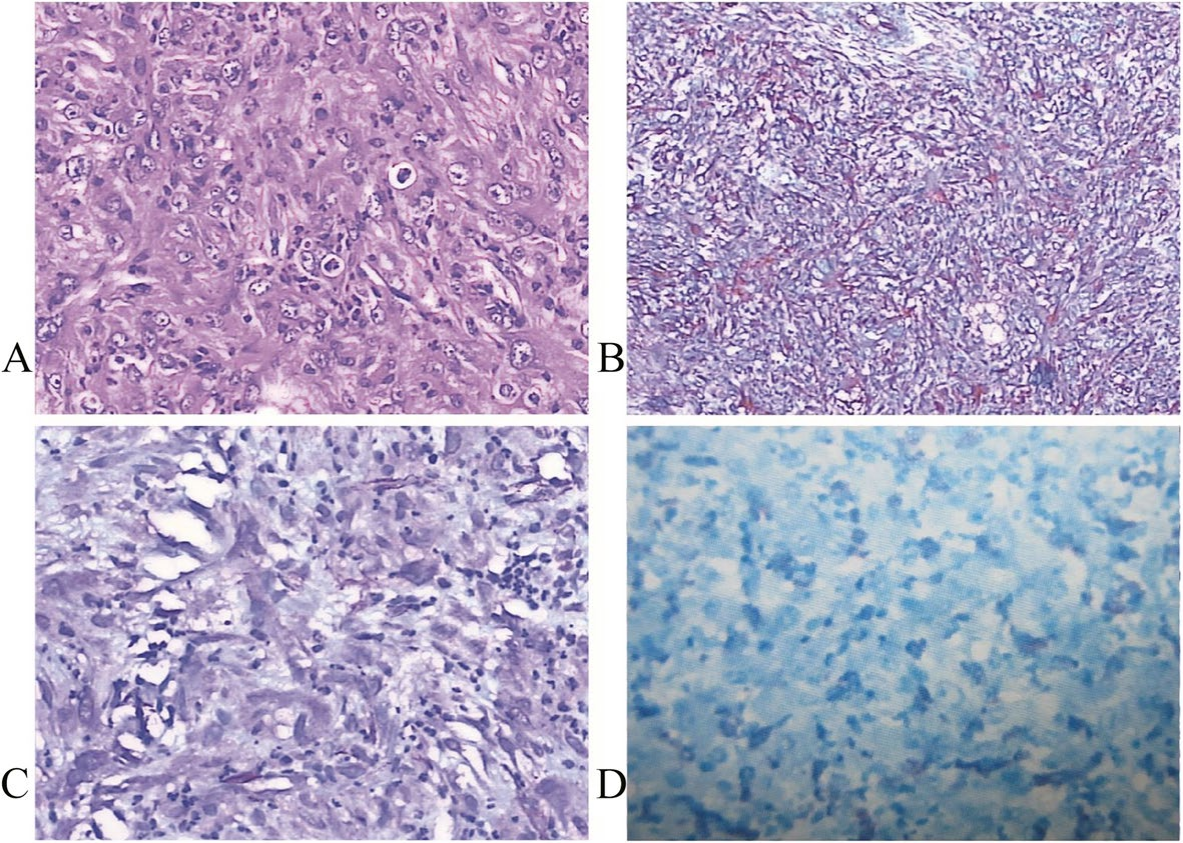
Photomicrograph by hematoxylin–eosin (HE) and immunohistochemistry staining for tumor. Photomicrograph showing proliferation of eosinophilic spindle cells with numerous inflammatory cells including lymphocytes and few granulocytes. [HE, original magnification, × 200] (**A**). Immunohistochemistry revealed tumor cell immunoreactivity for vimentin (**B**), smooth muscle actin (SMA) (**C**), and CD68 (**D**) (original magnification, × 200)
